# The Heidenhain variant of Creutzfeldt-Jakob disease

**DOI:** 10.1590/0100-3984.2017.0166

**Published:** 2019

**Authors:** Bernardo Carvalho Muniz, Lana Sayuri Makita, Bruno Niemeyer de Freitas Ribeiro, Edson Marchiori

**Affiliations:** 1 Instituto Estadual do Cérebro Paulo Niemeyer - Departamento de Radiologia, Rio de Janeiro, RJ, Brazil.; 2 Universidade do Estado do Rio de Janeiro (UERJ), Rio de Janeiro, RJ, Brazil.; 3 Universidade Federal do Rio de Janeiro (UFRJ), Rio de Janeiro, RJ, Brazil.

**Figure 1:**
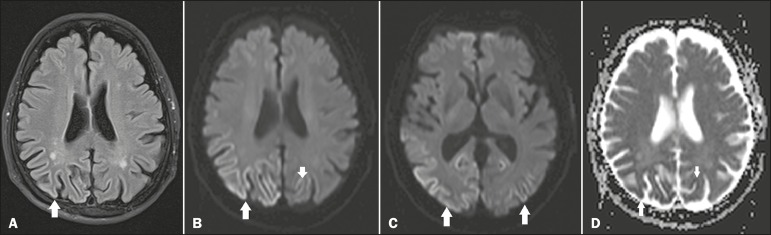
**A:** Axial FLAIR MRI sequence showing a hyperintense signal in the bilateral parieto-occipital cortex (arrow), more evident on the right, sparing the subcortical white matter. **B:** Axial diffusion-weighted MRI, at the same level depicted in **A**, showing restricted diffusion in the parieto-occipital cortex (arrow). **C:** Axial diffusion-weighted MRI, at the level of the basal ganglia and thalami, showing no changes in signal intensity. Note the restricted diffusion in the bilateral parietooccipital cortex (arrows). **D:** Axial MRI, with apparent diffusion coefficient mapping, at the levels depicted in **A** and **B**, showing low signal intensity, confirming the restricted diffusion, in the cortical lesions.

Dear Editor,

A 78-year-old man presented with a two-month history of progressive spatial disorientation and altered color perception, without significant behavioral changes or seizures. An ophthalmologic examination showed no alterations. Serological tests for HIV and syphilis were negative. On magnetic resonance imaging (MRI) of the brain, fluid-attenuated inversion recovery (FLAIR) sequences showed a hyperintense signal in the cortical region, most pronounced in the parietal and occipital lobes, together with restricted diffusion (Figure 1). There were no signs of involvement of the white matter or basal ganglia; nor was there any contrast enhancement. A diagnosis of Heidenhain variant of Creutzfeldt-Jakob disease (HvCJD) was suggested, and that hypothesis was corroborated by electroencephalography, which showed acute, periodic triphasic waves, predominantly in the posterior areas.

CJD, also known as transmissible spongiform encephalopathy or prion disease, is a rare, rapidly progressive neurodegenerative disease with no predilection for gender, preferentially affecting patients between the fifth and eighth decades of life. It can be sporadic, which is the most common form, accounting for 85% of cases; inherited, by various mutations in the prion protein gene; iatrogenic, caused by inoculation of prions with contaminated materials; or in a variant form, which usually results from the transmission of bovine spongiform encephalopathy to humans, usually through the consumption of contaminated meat^(^^1^^-^^3^^)^. The typical clinical findings include a rapid decline in cognitive function, followed by myoclonic jerks and akinetic mutism. However, in HvCJD, the classic manifestation is cortical blindness due to involvement of the parieto-occipital cortex, which can be accompanied by myoclonus and progressive dementia^(^^1^^,^^3^^)^.

MRI studies have come to play an ever more important role in the evaluation of patients with neurological diseases^(^^4^^-^^7^^)^. On MRI, the sporadic and inherited forms of CJD usually present areas of high signal intensity in T2-weighted and FLAIR sequences, with restricted diffusion, in the cerebral cortex and the basal ganglia, especially the striatum, in a focal or diffuse, symmetric or asymmetric form, sparing the region around the rolandic cortex and the thalami^(^^3^^)^. Classic signs such as the pulvinar sign and the "hockey stick" sign are typical of the variant form and are characterized respectively by hyperintense signals in T2-weighted and FLAIR sequences of the posterior and posteromedial thalami^(^^8^^,^^9^^)^.

In HvCJD, there is invariably involvement of the parieto-occipital cortex, including the primary visual cortex, characterized on MRI by hyperintense signals in T2-weighted and FLAIR sequences, together with restricted diffusion, typically with preservation of the subcortical white matter and of the basal ganglia. It is noteworthy that restricted diffusion can precede the clinical manifestations of CJD^(^^3^^)^.

In HvCJD, the electroencephalogram typically shows acute, periodic triphasic waves, predominantly in the posterior areas^(^^10^^)^. Analysis of the cerebrospinal fluid can reveal elevated 14-3-3 protein levels^(^^3^^)^. Histopathological analysis is the gold standard diagnostic method, showing marked neuronal loss, spongiform changes, intense astrogliosis and immunoreactivity to the abnormal pathogenic isoform of the prion protein^(^^11^^)^. The prognosis is bleak, and death usually occurs within one year^(^^2^^,^^9^^)^.

It is important to make the differential diagnosis of HvCJD. The main differential diagnoses are frontotemporal dementia, status epilepticus, hypoxic-ischemic encephalopathy, severe hypoglycemia, immune-mediated autoimmune encephalopathy, posterior cortical atrophy, and hyperammonemia^(^^3^^)^. Although rare, HvCJD should be borne in mind in the differential diagnosis of visuospatial deficits, especially when MRI shows areas of high signal intensity in T2-weighted and FLAIR sequences, together with restricted diffusion, in the cortical region of the occipital lobes.
